# Abiotic Stresses Downregulate Key Genes Involved in Nitrogen Uptake and Assimilation in *Brassica juncea* L.

**DOI:** 10.1371/journal.pone.0143645

**Published:** 2015-11-25

**Authors:** Parul Goel, Anil Kumar Singh

**Affiliations:** 1 CSIR-Institute of Himalayan Bioresource Technology, Palampur-176 061 (HP), India; 2 Academy of Scientific and Innovative Research, New Delhi, India; CSIR-National Botanical Research Institute, INDIA

## Abstract

Abiotic stresses such as salinity, drought and extreme temperatures affect nitrogen (N) uptake and assimilation in plants. However, little is known about the regulation of N pathway genes at transcriptional level under abiotic stress conditions in *Brassica juncea*. In the present work, genes encoding nitrate transporters (NRT), ammonium transporters (AMT), nitrate reductase (NR), nitrite reductase (NiR), glutamine synthetase (GS), glutamate synthase (GOGAT), glutamate dehydrogenase (GDH), asparagines synthetase (ASN) were cloned from *Brassica juncea* L. var. Varuna. The deduced protein sequences were analyzed to predict their subcellular localization, which confirmed localization of all the proteins in their respective cellular organelles. The protein sequences were also subjected to conserved domain identification, which confirmed presence of characteristic domains in all the proteins, indicating their putative functions. Moreover, expression of these genes was studied after 1h and 24h of salt (150 mM NaCl), osmotic (250 mM Mannitol), cold (4°C) and heat (42°C) stresses. Most of the genes encoding nitrate transporters and enzymes responsible for N assimilation and remobilization were found to be downregulated under abiotic stresses. The expression of *BjAMT1*.*2*, *BjAMT2*, *BjGS1*.*1*, *BjGDH1* and *BjASN2* was downregulated after 1hr, while expression of *BjNRT1*.*1*, *BjNRT2*.*1*, *BjNiR1*, *BjAMT2*, *BjGDH1* and *BjASN2* was downregulated after 24h of all the stress treatments. However, expression of *BjNRT1*.*1*, *BjNRT1*.*5* and *BjGDH2* was upregulated after 1h of all stress treatments, while no gene was found to be upregulated after 24h of stress treatments, commonly. These observations indicate that expression of most of the genes is adversely affected under abiotic stress conditions, particularly under prolonged stress exposure (24h), which may be one of the reasons of reduction in plant growth and development under abiotic stresses.

## Introduction

**Fig 1:**
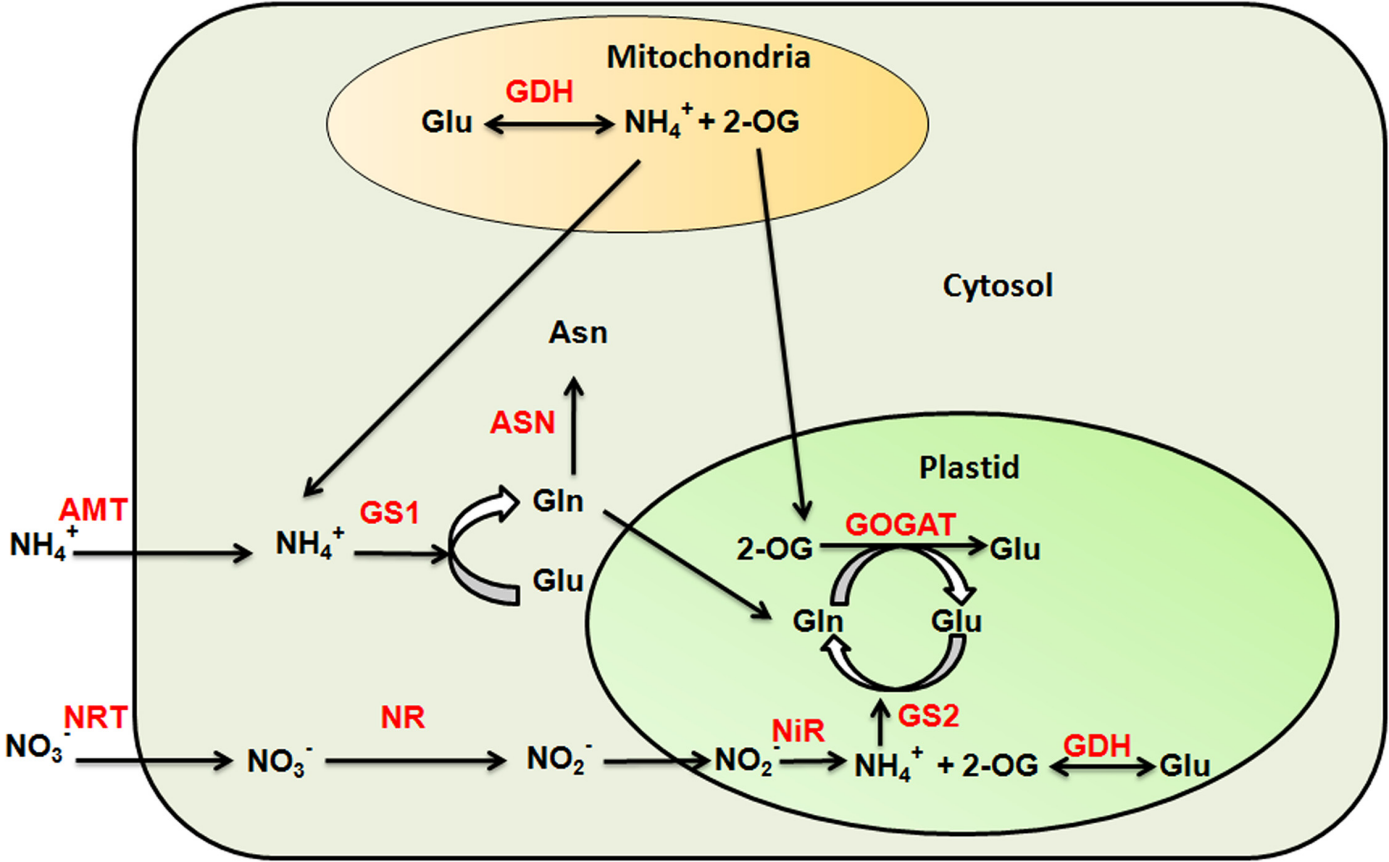
Nitrogen uptake and assimilation process in plants. The uptake of nitrate (NO_3_^-^) and ammonium (NH_4_^+^) ions is mediated by nitrate (NRT) and ammonium transporters (AMT), respectively. The NO_3_^-^ entered into the cell is reduced to nitrite ions (NO_2_^-^) by an enzyme nitrate reductase (NR). The nitrite ion then moves to plastid and reduced to ammonium ion by nitrite reductase (NiR) enzyme. The ammonium is then incorporated into amino acid by glutamine synthetase and glutamate synthase via GS/GOGAT cycle. The ammonium ion transported by ammonium transporters directly enters into GS/GOGAT cycle. The two additional enzymes glutamate dehydrogenase (GDH) and asparagine synthetase (ASN) also participates in ammonium assimilation. The GS, GDH and ASN are the key enzymes involved in synthesis of glutamine (Gln), Glutamate (Glu) and Asparagine (Asn).

Nitrogen (N) is a primary plant nutrient that plays a crucial role in determining plant growth and productivity. Plants require nitrogen for the synthesis of vital molecules, such as proteins, nucleic acids and chlorophyll. Most plant species are capable to absorb and assimilate nitrate (NO_3_^-^) and ammonium (NH_4_^+^). In plants uptake of nitrate and ammonium is an active process mediated by nitrate transporters (NRT) and ammonium transporters (AMT), respectively. The nitrogen assimilation involves reduction of nitrate to ammonium which is finally incorporated into amino acids by the process of ammonia assimilation (Fig 1). In plants, several processes, including N uptake and assimilation are known to be adversely affected by abiotic stresses, such as salinity, drought, and extreme temperatures. The uptake of nitrogen, its translocation from root to shoot and finally its assimilation has been found to be affected by high salinity in cowpea [1]. Nitrogen use efficiency (NUE) was also reported to be reduced significantly with increased salinity conditions in chile pepper [2]. During high salt stress, Na^+^ ions disrupt the membrane integrity of plant roots by displacing the Ca^2+^ ions that maintains the integrity of the membrane [3]. The high salinity has been shown to inhibit the activity of many enzymes involved in nitrogen assimilation in maize, mung bean and tomato [4–7]. Similarly under drought stress, activities of nitrate reductase (NR) and glutamine synthetase (GS) were found to be reduced in barley [8]. In case of wheat, drought stress was shown to limit the nitrogen translocation during grain filling, resulting in to low grain yield [9]. High temperature has also been shown to inhibit the nitrate uptake and assimilation in creeping bentgrass, wheat and rice [10–13].

*Brassica juncea* is an important oilseed crop worldwide. Increased level of salinity was found to reduce the activities of various enzymes involved in nitrogen assimilation, *viz*. NR, NiR, GS, GOGAT and GDH in *B*. *juncea* [14,15]. Salinity stress was also found to reduce the biomass, shoot and root length and CO_2_ assimilation rate in *B*. *juncea* [16]. High temperature was found to adversely affect certain morpho-physiological parameters such as leaf area index, crop growth rate, chlorophyll stability, abortion of flower and also reduces the nitrogen, phosphorous and potassium content in *B*. *juncea* [17–20]. Drought stress was also found to adversely affect both dry matter and seed yield of mustard and canola [21]. Effect of drought stress on twenty two advanced breeding lines of *B*. *juncea* was studied, which showed that the process of photosynthesis, transpiration, water use efficiency and other morpho-physiological characters were adversely affected [22]. The effect of drought on yield and yield components was also investigated on fourteen different genotypes of Indian mustard under irrigated and rain fed conditions, which showed that plant height, primary and secondary branches per plant and seed yield were inhibited [23]. Most of these studies have been done to study the effect of abiotic stresses on morphological and physiological characteristics.

Several transgenics overexpressing genes of N pathway have shown improved tolerance against abiotic stresses. Transgenic tobacco overexpressing NR gene showed retention of 50% NR activity under drought stress as compared to untransformed plants, where NR activity was not detected [24]. Overexpression of chloroplast *GS2* has resulted in enhanced salt tolerance in transgenic rice [25]. Ectopic overexpression of pine cytoplasmic glutamine synthetase (*GS1*) in transgenic poplar showed enhanced tolerance to drought stress [26]. However, overexpression of *OsGS1;2* gene in rice conferred higher sensitivity to salt, drought and cold stress [27]. The overexpression of *OsGS* gene in transgenic rice enhanced tolerance to cadmium stress by modulating the oxidative stress response [28]. Overexpression of *E*. *coli gdhA* conferred tolerance under water deficit conditions in transgenic tobacco and maize [29–31]. These studies clearly showed that by improving the nitrogen use efficiency of plants, optimum plant growth and productivity may be obtained under stress condition. However, little is known about the regulation of N pathway genes at transcriptional level under abiotic stress conditions. In the present study, we have cloned twenty six genes encoding nitrate and ammonium transporters and enzymes involved in N assimilation from *B*. *juncea* and carried out their expression profiling under salt, osmotic, cold and heat stresses after 1h and 24h of treatment. Our results showed that expression of some key genes involved in N-uptake and assimilation was downregulated under abiotic stress conditions.

## Materials and Methods

### Plant material, growth conditions and stress treatments

Seeds of *B*. *juncea* cv. Varuna were kindly provided by Prof. Deepak Pental, Department of Genetics, Delhi University South Campus, New Delhi, India. Healthy seeds were surface sterilized with 70% ethanol for 2–3 minutes followed by repeated washing with autoclaved distilled water and allowed to germinate over sterile seed germination sheets in dark for 3 days and then transferred to light. The one weak-old seedlings were transferred to ½ strength Murashige and Skoog medium (MS medium) [32] and acclimatized in control conditions for 3–4 hours. For stress imposition, the plants were treated with ½ strength MS medium supplemented with 150mM NaCl or 250mM mannitol or seedlings were exposed to 4°C or 42°C for 1hr and 24hr duration. Plants treated with ½ strength MS medium grown under control conditions were considered as untreated control. Each treatment had three replicates. Following stress treatments, the plants were washed with distilled water; tissues were harvested, wrapped in aluminium foil, frozen in liquid nitrogen and then stored in -80°C until future use.

### Cloning and sequence analysis

Out of various genes involved in N uptake and assimilation only one gene encoding for high-affinity nitrate transporter (Accession no. AEZ68614.1) of *B*. *juncea* was available in NCBI database as on July 31, 2014. Since whole genome of *B*. *juncea* has not been sequenced, coding DNA sequences of NRT1, NRT2, AMT, NR, NiR, GS, GOGAT, GDH and ASN of *Arabidopsis*, *B*. *napus* and *B*. *rapa* were downloaded from the *Arabidopsis* information resource (TAIR version 10) and NCBI database (http://www.ncbi.nlm.nih.gov/). Degenerate primers (S1 File) were designed to amplify full length coding sequences using cDNA of *B*. *juncea* cv. Varuna seedlings as a template. The PCR products were cloned in pGEMT Easy cloning vector (Promega) and confirmed by nucleotide sequencing. All the cloned cDNAs have been submitted to GenBank and the accession numbers have been shown in Table 1. The isolated sequences were BLAST searched against NCBI database in order to identify the homologous sequences. The nucleotide sequences were translated using Expasy (http://www.expasy.org/). The molecular weight and isoelectric point of predicted proteins were obtained using Compute PI/MW tool of Expasy. The sub-cellular localizations of translated proteins were predicted using WoLF-PSORT program and Cell-PLoc 2.0 program [33–34]. Conserved domains in various proteins were identified using PFAM database (http://pfam.xfam.org/). The amino acid sequence similarity was also checked among the homologs using BLASTP. To perform phylogenetic analysis of protein sequences with *A*. *thaliana* orthologs, the amino acid sequences were aligned using clustalw and phylogenetic trees were made by neighbour-joining method with 1000 bootstrap replicates using MEGA6 software [35].

**Table 1:** List of proteins along with their molecular weight (M. wt.), isoelectric point (PI), CDS and protein length, subcellular localization and *Arabidopsis thaliana* orthologs.

Protein	Accession numbers	M. wt. (KDa)	PI	CDS length (bp)	Protein length (aa)	Subcellular localization	At ortholog locus ID	At protein description	Bit score
BjNRT1.1	KT119578	64.97	8.57	1767	589	Plasma membrane	AT1G12110	nitrate transporter 1.1/AtNPF6.3	1013
BjNRT1.2	KT119579	63.84	8.79	1743	581	Plasma membrane	AT1G69850	nitrate transporter 1:2/AtNPF4.6	960
BjNRT1.3	KT119580	65.17	8.99	1764	588	Plasma membrane	AT3G21670	AtNPF6.4/ NRT1/PTR family 6.4	928
BjNRT1.4	KT119581	63.43	8.78	1731	577	Plasma membrane	AT2G26690	AtNPF6.2/ NRT1/PTR family 6.2	1003
BjNRT1.5	KT119582	69.49	5.7	1860	620	Plasma membrane	AT1G32450	AtNPF7.3/ nitrate transporter 1.5	1113
BjNRT1.7	KT119583	68.70	8.95	1866	622	Plasma membrane	AT1G69870	AtNPF2.13/ nitrate transporter 1.7	987
BjNRT1.8	KT119584	64.72	6.22	1749	583	Plasma membrane	AT4G21680	AtNPF7.2,/nitrate transporter 1.8	1043
BjNRT2.1	KT119585	51.19	9.2	1407	469	Plasma membrane	AT1G08090	ACH1/nitrate transporter 2:1	899
BjNRT2.7	KT119586	51.84	9.06	1455	485	Plasma membrane	AT5G14570	AtNRT2.7, high affinity nitrate transporter 2.7	647
BjAMT1.1	KT119596	53.64	7.11	1509	503	Endoplasmic reticulum	AT4G13510	ammonium transporter 1;1	822
BjAMT1.2	KT119597	54.57	8.04	1530	510	Plasma membrane	AT1G64780	ammonium transporter 1;2	823
BjAMT2	KT119598	52.532	7.12	1464	488	Plasma membrane	AT2G38290	ammonium transporter 2	825
BjNR1	KT119587	102.25	6.6	2730	910	Chloroplast	AT1G77760	nitrate reductase 1	1698
BjNR2	KT119588	102.41	6.23	2733	911	Cytopalsm	AT1G77760	nitrate reductase 1	1675
BjNiR	KT119589	65.40	6.29	1755	585	Chloroplast	AT2G15620	nitrite reductase/NiR1	1106
BjGS1.1	KT119590	39.163	5.28	1068	356	Cytopalsm	AT5G37600	glutamine synthase 1;1	707
BjGS1.3	KT119591	38.59	6.4	1062	354	Cytopalsm	AT3G17820	glutamine synthase 1;3	624
BjGS2	KT119593	47.37	5.84	1284	428	Chloroplast	AT5G35630	glutamine synthetase 2	821
BjFd-GOGAT	KT119603	154.66	6.35	4806	1602	Chloroplast	AT5G04140	ferredoxin-dependent glutamate synthase	3022
BjNADH-GOGAT	KT119604	240.68	6.05	6597	2199	Chloroplast	AT5G53460	nadh-dependent glutamate synthase 1	3979
GDH1	KT119594	44.61	6.23	1233	411	Chloroplast	AT5G18170	glutamate dehydrogenase 1	814
GDH2	KT119595	35.13	5.78	984	328	Mitochondria	AT5G07440	glutamate dehydrogenase 2	658
ASN1	KT119599	64.52	5.92	1722	574	Cytopalsm	AT3G47340	glutamine-dependent asparagine synthase 1	1076
ASN2	KT119600	63.86	5.98	1695	565	Cytoplasm	AT5G65010	asparagine synthetase 2	1045
ASN3	KT119601	65.33	6.25	1740	580	Cytoplasm	AT5G10240	asparagine synthetase 3	1024
ASN4	KT119602	42.38	6.98	1128	376	Chloroplast	AT2G03667	asparagine synthase family protein	674

### RNA isolation and quantitative real-time PCR

Total RNA was extracted from all samples using iRIS method [36]. The concentration and quality of RNA were checked using NanoDrop 1000. The total RNA was treated with DNase I (Fermentas Life Sciences, USA) at 37°C for 30 minutes to degrade any DNA contamination in RNA sample. For cDNA synthesis, 3μg of total RNA was reverse transcribed using Revert-Aid H Minus Reverse Transcriptase kit (Thermo Scientific, USA) in a final volume of 20 μL according to the manufacturer’s instructions and cDNA was diluted 10 times in nuclease free water. The qRT-PCR analysis was performed using gene specific primers designed using PrimerExpress software version 3.0.1 (Applied Biosystems; S2 File). Proper amplification for *BjASN3* and *BjASN4* was not obtained, thus not included in expression analysis. The qRT-PCR reactions were performed on Step One real-time PCR machine (Applied Biosystems, USA). Each reaction contained 2.5μL diluted cDNA, 10mM each of gene specific forward and reverse primers and 5 μL of SYBR Green qPCR Master Mix (Applied Biosystems, USA) in a final volume of 10μL. qRT-PCR was done in three technical and three biological replicates. The following thermal profile was used for PCR amplification: 95°C for 10 min followed by 40 cycles of 95°C for 15s and 60°C for 1 min. The specificity of reactions was checked by melting curve analysis. The *B*. *juncea* ubiquitin gene *UBQ9* was used as an internal control for normalization of relative mRNA abundance as suggested [37]. The qRT-PCR reactions were performed with three biological and three technical replicates. The relative expression ratios of target genes with respective controls were calculated using REST 2009 software (Qiagen).

### Statistical analysis of the data

The genes were considered to be significantly up or down-regulated if the change in expression was ≥ 2 fold or <0.5 fold and p-value <0.05 (Student’s t-test). The mean value of relative expression with respect to control was used to plot figures and error bars represent standard error of three biological replicates. To identify statistical significance in the expression of genes between two time points, Student’s t-test was used with p-value <0.05; p-value <0.001; p-value <0.0001.

## Results and Discussion

### Cloning of genes involved in nitrogen uptake and assimilation

**Fig 2:**
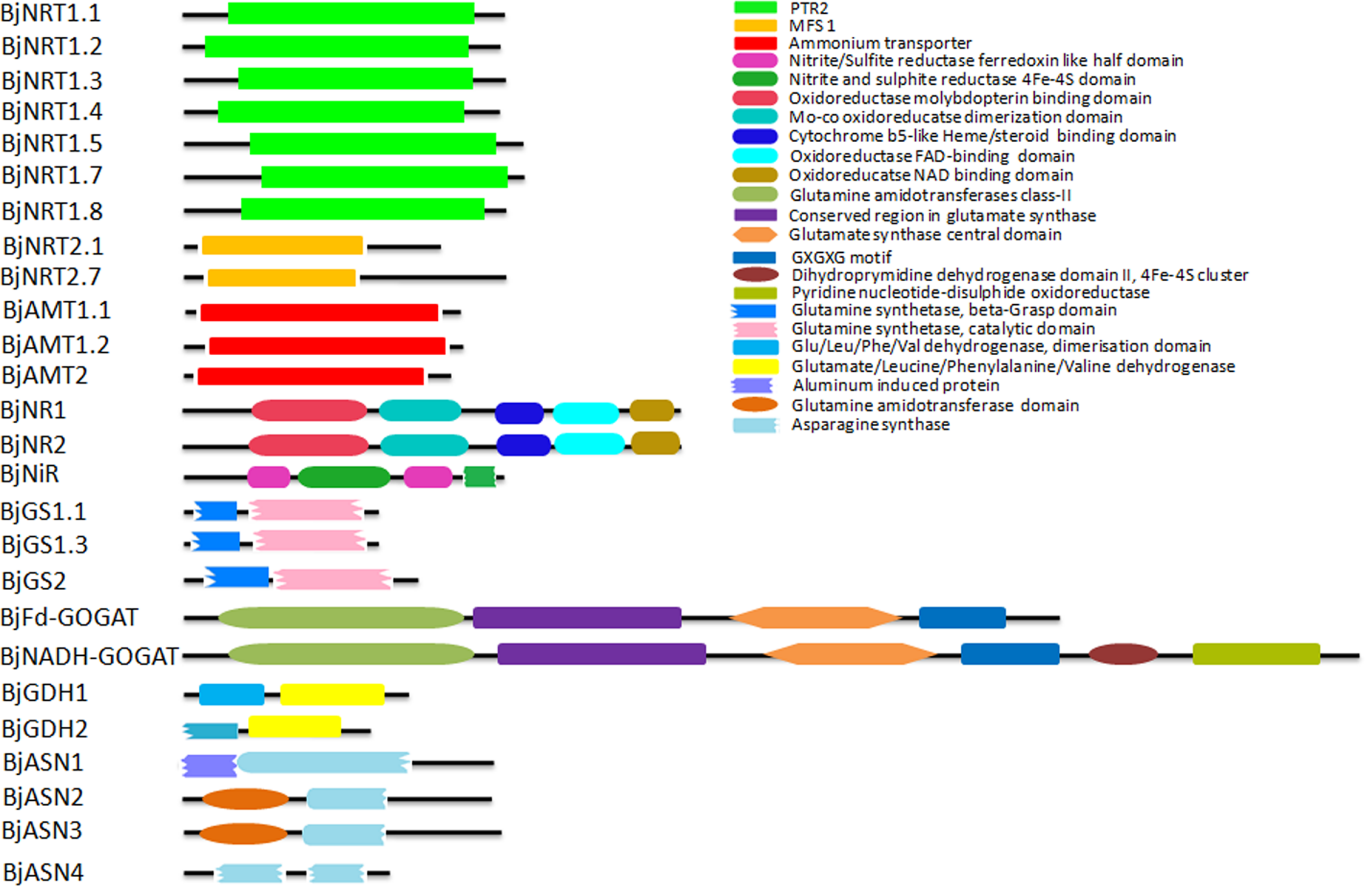
The conserved domain architecture of various proteins involved in nitrate and ammonium uptake, nitrate reduction and ammonia assimilation in *B*. *juncea*.

Full-length CDSs of various genes amplified using cDNA prepared from total RNA isolated from seedlings of *B*. *juncea* cv. Varuna were cloned and their sequences confirmed through nucleotide sequencing. The sizes of predicted proteins ranged between 328–2199 amino acids. The molecular weight and PI of the predicted proteins ranged between 35.13 KDa-240.6 KDa and 5.28–9.2, respectively (Table 1). The conserved domain analysis revealed that all the NRT1 proteins contain a single proton-dependent oligopeptide transporter (PTR; PF00854) domain and NRT2 proteins contain major facilitator superfamily domain (MFS_1; PF07690) domain (Fig 2). Members of the AMT family were found to contain a single ammonium transporter domain (PF00909). The NR proteins contain 5 major domains, namely Mo_Co dimer (PF03404), Oxidored molyb (PF00174), FAD binding_6 (PF00173), NAD binding _1 (PF00970) and 1cyt_b5 (PF00175). The NiR proteins contain two NIR_SIR_Ferr (PF03460) and NIR_SIR domains (PF01077). The GS proteins contain two domains, Gln_syn_N (PF03951) and Gln_syn_C (PF00120). The Fd-GOGAT protein contains four domains GTase_2 (PF00310), Glu_syn_central (PF04898), Glu _synthase (PF01645) and GXGXG (PF01493) domain however NADH-GOGAT contain two additional domains Fer_4_20 (PF14691) and pyr_redox_2 (PF07992).The GDH proteins contain two domains, namely ELFV_dehydrog_N (PF02812), ELFV_dehydrog (PF00208). The ASN1 protein contains two domains, namely DUF3700 (PF12481) and Asn_synthase (PF00733), while ASN2, ASN3 and ASN4 proteins contain GATase_7 (PF13537) and Asn_synthase domain (PF00733). To determine the phylogenetic relationship of *B*. *juncea* proteins with that of their *A*. *thaliana* orthologs, phylogenetic trees were made (S1 Fig). The *B*. *juncea* proteins and their respective *Arabidopsis* orthologs were found to be clustered together.

The prediction of subcellular localization has shown that most of the proteins were predicted to be localized in the organelles of their site of action (Table 1). For e.g., all the BjNRT1 and BjNRT2 transporters were predicted to be localized in plasma membrane. The BjAMT1.2 and BjAMT2 transporters were also predicted to be localized in the plasma membrane, while BjAMT1.1 was predicted to be localized in endoplasmic reticulum (ER). Similarly in wild grass *Puccinellia tenuiflora*, PtAMT1.1 was also shown to be localized in endomembrane [38]. The BjNR and BjNiR were found to be localized in cytoplasm and chloroplast, respectively where actual processes of nitrate and nitrite reduction takes place [39]. The two isoforms of glutamine synthetase, BjGS1 and BjGS2 involved in ammonia assimilation were predicted to be localized in cytoplasm and chloroplast [40]. The BjFd-GOGAT and BjNADH-GOGAT both were predicted to be localized in chloroplast, the main organelle where GS/GOGAT cycle takes place [41].The BjGDH1 and BjGDH2 were predicted to be localized in chloroplast and mitochondria, respectively. Similarly, GDH1 in *Chlorella* and GDH2 in *Vitis vinifera* were found to be localized in chloroplast and mitochondria, respectively [42,43]. The BjASN1, BjASN2 and BjASN3 proteins involved in asparagine synthesis were predicted to be localized in cytosol [44]. Whereas, BjASN4 was found to be localized in chloroplast.

### Effect of abiotic stresses on expression of genes encoding transporters

In plants, short term and long term exposure to stress leads to change in gene expression for cellular adaptation to changing environment [45]. In present study, expression of all the genes involved in N uptake and assimilation was studied following 1h and 24h of abiotic stress exposure. The change in gene expression after 1h provides insights into the plant acclimation to an early stress and can be considered as a general response to stress conditions. Whereas, transcriptional changes have been found to become more specific to abiotic stress after prolonged stress exposure [46]. Therefore, expression changes after 24h may reveal genes that are more specific to abiotic stress under longer duration.

#### Nitrate transporters

**Fig 3:**
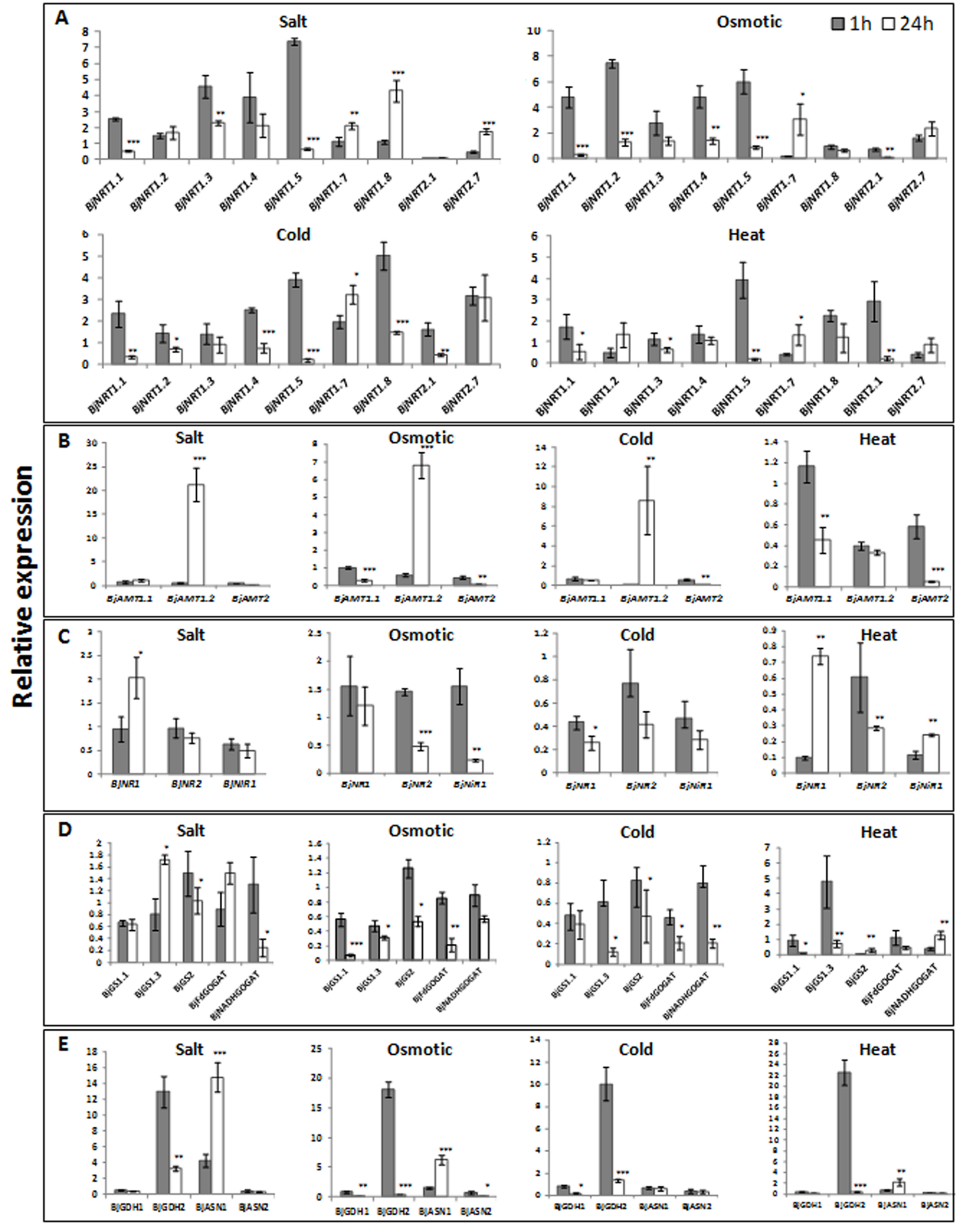
Bar diagrams showing relative expression of various genes encoding (A) nitrate and (B) ammonium transporters and enzymes involved in (C) nitrate and nitite reduction and (D,E) ammonium assimilation in *B*. *juncea* under abiotic stress conditions after 1h (grey bars) and 24h (white bars) as compared to untreated control plants. Relative expression ratios were determined using qRT-PCR. Asterisks on the top of the bars indicate statistically significant differences (* p-value<0.05 (significant), **p-value < .01(highly significant), ***p < .001 (very highly significant) between 1h and 24h stress treated samples.

The first step of NO_3_ assimilation is its uptake with the help of nitrate transporters located on the plasma membrane of the root epidermal and cortical cells. In higher plants, two types of nitrate transporters have been found, low affinity nitrate transporters (NRT1) and high affinity nitrate transporters (NRT2). The amino acid sequence identity among seven NRT1 homologs ranges between 30.1–68.3%, while two NRT2 homologs share 46% sequence identity (S3 File). Expression analysis of 7 genes encoding NRT1 (*BjNRT1*.*1*, *BjNRT1*.*2*, *BjNRT1*.*3*, *BjNRT1*.*4*, *BjNRT1*.*5*, *BjNRT1*.*7*, *BjNRT1*.*8*) and 2 genes encoding NRT2 (*BjNRT2*.*1*, *BjNRT2*.*7*) was carried out under various abiotic stresses (Fig 3A, S2 Fig). The expression of *BjNRT1*.*1* and *BjNRT1*.*5* was found to be upregulated after 1h of all the stress treatments, while their expression was either downregulated or remained unaltered under 24h of the treatments. In *Arabidopsis*, CHL1 (*AtNTR1*.*1*) was shown to function as dual-affinity nitrate transporter contributing to both low and high affinity nitrate uptake in *Arabidopsis* roots [47], while NRT1.5 is mainly involved in long distance transport of nitrate from root to shoot [48]. In plants, early response to environmental stresses is critical to ensure cell survival. Therefore, initial upregulation of these genes might reflect their importance in plant adaptation to various stresses. In plants, root is the first tissue to perceive stress signal and repression in lateral root number and growth has been well established as an adaptive response under stress conditions, like salt and drought [49,50]. In *Arabidopsis*, NRT1.1 was found to accelerate lateral root growth in nitrate rich patches of external medium by accumulating auxin in the lateral root tip [51]. The downregulation of *BjNRT1*.*1* after 24h of stress exposure might participate in altering root morphology that may in turn help plant to withstand stress conditions. In normal conditions, majority of the nitrate is transported from root to shoot for assimilation process but under stress conditions the reallocation of nitrate to root was observed [52,53]. In *A*. *thaliana*, this reallocation due to downregulation of *AtNRT1*.5 has been found to be involved in stress tolerance mechanism [54–55]. In present study, the downregulation of *BjNRT1*.5 after 24h suggests its involvement in plant tolerance mechanism to abiotic stresses. The expression of *BjNRT1*.*2* was downregulated at 24h of cold and 1h of heat stress treatments, while it remained unaltered under rest of the conditions. In Poplar, differential expression of NRT1 and NRT2 genes in response to phytohormones and ion stress has been correlated with difference in *cis*-regulatory elements in their promoter regions [56]. The expression of *BjNRT1*.*3* was upregulated under salt stress (1h, 24h) and under osmotic (1h) treatment, while under rest of the conditions, its expression remained unchanged. In rice, expression of *OsNRT1*.*3* gene was upregulated under drought, while treatment with ABA and NaCl did not affect its expression [57]. The expression of *BjNRT1*.*4* was upregulated under salt (1h, 24h), osmotic (1h) and cold (1h) stresses, while it was found to be downregulated under cold (24h) and remained unchanged under rest of the conditions. The *BjNRT1*.*7* was found to be the only gene, expression of which was upregulated after 24h of all the stress treatments. The expression of *BjNRT1*.*8* was upregulated under salt (24h), cold (1h) and heat (1h) stresses, while in rest of the conditions, its expression was either downregulated or remained unaltered. The expression of *AtNRT1*.*8* was found to be strongly upregulated in *Arabidopsis* roots under cadmium stress [55]. The expression of *BjNRT2*.*1* was downregulated under all the stress conditions, except cold (1h) where it was unaltered and under heat (1h) where it was upregulated. The downregulation of *BjNRT2*.1, which is a member of high affinity nitrate transporter (HATS) family, may be due to the fact that all the stress treatments were given in ½ strength MS medium, where nitrate concentration is 19.7mM. It is well known that members of HATS play role in nitrate uptake when external nitrate concentration is very low (<250μM) [58]. Exposure to stress may further lead to downregulation of *BjNRT2*.*1*. Downregulation of *AtNRT2*.*1* gene under salt stress was also reported in tomato [49]. In *Arabidopsis*, AtNRT2.1 was shown as an important component of inducible high-affinity nitrate transport system (IHATS) and its disruption in *Atnrt2*.*1* mutant led to 72% reduction in IHATS [59]. These observations suggest that downregulation of two important nitrate transporter genes, *BjNRT1*.*1* and *BjNRT2*.*1* under abiotic stress conditions disrupts both low- and high-affinity nitrate transport systems, which may be one of the key factors, inhibiting growth and development of *B*. *juncea* under abiotic stress conditions.

#### Ammonium transporters

In most of the natural environmental conditions, ammonium and nitrate are considered to be the main source of nitrogen for the plants growth. Uptake of ammonium ions by plants is more rapid process over nitrate ions when both of these are provided with equal concentration [60]. The BjAMT1.1 and BjAMT1.2 share 94% percent amino acid sequence identity (S3 File). Expression of two *AMT1* genes and one *AMT2* gene was studied under abiotic stress conditions. Expression of *BjAMT1*.*2* was found to be downregulated under 1h of salt, osmotic and cold stresses, while under 24h treatment of these stresses induced its expression (Fig 3B, S2 Fig). Under heat stresses, expression of *BjAMT1*.2 was found to be downregulated at both the time points (1h and 24h). The expression of *BjAMT1*.*1* was downregulated under all the treatments, except salt (24h), osmotic (1h) and heat (1h), where it remained unaltered. The expression of *BjAMT2* was found to be downregulated under all the stress conditions. Transcriptomics study of *Lotus japonicus* has revealed the downregulation of *AMT1* gene under drought stress [61]. In plants uptake of ammonium ion is energetically less expensive process as plants do not have to spend extra energy for reducing nitrate into ammonium. Our result suggested that both, nitrate and ammonium uptake are inhibited under stress conditions.

### Effect of abiotic stresses on expression of genes encoding enzymes involved in N assimilation

#### Nitrate and Nitrite reductases

Once the nitrate enters into the cytoplasm, it is reduced to nitrite with the help of nitrate reductase (NR). The nitrite is then transported into chloroplast and reduced into ammonium ion with the help of nitrite reductase (NiR). These enzymes have been shown to be highly regulated at transcriptional and translational level and also influenced by environmental factors [62]. The BjNR1 and BjNR2 were found to share 91% amino acid sequence identity (S3 File). The expression of *BjNR1* was found to be upregulated only under salt stress at 24h (Fig 3C, S2 Fig). In all other conditions, expression of *BjNR1* was found to be downregulated. Expression of *BjNR2* was found to be downregulated under all the stresses at 24h except, salt stress, while it remained unchanged under all the stresses at 1h. Similarly, expression of *BjNiR1* was downregulated under all the stress conditions, except osmotic stress at 1h (Fig 3C, S2 Fig). As expression of nitrate assimilation genes is controlled by nitrate content in plants [63], a severe downregulation of *BjNR2* and *BjNiR1* can be correlated with reduction in nitrate transport by roots under stress conditions. Reduced NR activity under salinity stress was reported in several plants, like barley, maize and tomato [64–66]. The reduction in NR activity under salt stress may be either due to direct effect of Cl^−^ions in the external medium causing reduction in nitrate uptake or may be due to low content of NR protein [67–69]. However increased expression of NR2 gene was reported in *Arabidopsis* roots during first week when treated with 100mM NaCl [70]. In leaves of winter wheat, activation of NR under short term low temperature stress was observed [71]. Repression of nitrate and nitrite reductase activity under osmotic and heat stress was also reported [72,73].

#### Glutamine synthetase and glutamate synthase

Glutamine synthetase (GS) is an ATP dependent enzyme that fixes the ammonium into glutamate to form glutamine. In plants, generally two types of GS proteins are present, GS1 and GS2 which are cytoplasmic and chloroplastic, respectively. The BjGS1.1 and BjGS1.3 were found to share 86.12% sequence identity at amino acid level (S3 File). Expression of *BjGS1*.*1*, *BjGS1*.*3* and *BjGS2* was studied under abiotic stresses (Fig 3D, S2 Fig). Expression of *BjGS1*.*1* was found to be downregulated under all the stress conditions. Similarly, expression of *BjGS1*.*3* was also found to be downregulated under all the stress conditions, except 1h of heat stress, where it was strongly upregulated. Expression of *BjGS2* was also downregulated under all the stress conditions, except salt (1h, 24h) and osmotic (1h) stress. The GS activity and its transcript accumulation was found to be affected by salt stress in an organ dependent manner, with increased ammonia assimilation in roots and decreased assimilation in leaves of potato plants [74]. Reduction in GS activity was already reported in tea bud under cold, drought and heat stresses [75]. Under stress conditions, deviation of glutamate from GS/GOGAT cycle to the synthesis of organic osmoticum has been observed [76]. Therefore reduction in the expression of glutamine synthetase gene under most of the stress might be due to low availability of glutamate. Reduced expression of GS gene may slow down process of ammonia assimilation and thus affects nitrogen metabolism. This suggested that abiotic stress severely affected the process of ammonia assimilation in *B*. *juncea*.

In plants, glutamate synthase mainly occurs in two forms, Fd-GOGAT and NADH-GOGAT. This enzyme catalyzes the transfer of the amide nitrogen of glutamine to 2-oxoglutarate to form two molecules of glutamate. In present study, *BjFd-GOGAT* and *BjNADH-GOGAT* were found to share 44% sequence identity at amino acid level (S3 File). The expression of *BjFd-GOGAT* was found to be downregulated after 24h of osmotic, cold and heat stress, whereas expression of *BjNADH-GOGAT* was downregulated after 24h of salt, osmotic and cold stress (Fig 3D, S1 Fig). In nodules of *Vicia faba*, activity of NADH-GOGAT was found to be decreased more than that of GS under 100mM NaCl stress [77,78]. The activity of GOGAT was also strongly inhibited in *Cicer arietinum* under 100 mM salt stress [79].

#### Glutamate dehydrogenase

Glutamate dehydrogenase enzyme catalyzes the reversible reaction involving assimilation of ammonia into glutamate and deamination of glutamate to form 2-oxoglutarate and ammonium. In plants, two types of GDH have been found, depending on the cofactor *viz*. NAD(H) dependent (mitochondrial) and NADP(H) dependent (chloroplast). Glutamate dehydrogenase is considered as an alternative enzyme for GS/GOGAT cycle under abiotic stresses [80]. Moreover, both aminating and deaminating properties of GDH were found to be helpful for plant during stress condition, as amination leads to detoxification of excess ammonium ions accumulated during stress [81] and deamination activity provides intermediate to the TCA cycle and thus sustained carbohydrate metabolism [82]. The expression of *BjGDH1* was downregulated under all the stress conditions, except 24h of cold stress, (Fig 3E, S2 Fig). The percent identity between BjGDH1 and BjGDH2 was found to be 76% (S3 File). However, expression of *BjGDH2* gene displayed a strong upregulation under both, 1h and 24h of salt stress, while under osmotic, cold and heat stresses, its expression was upregulated only at 1h time point. The GDH activity was found to be increased in salt tolerant rice cultivar in response to high NaCl concentration upto 800mM as compared to salt sensitive cultivar [82]. Increased expression of *GDH* under stress condition was also reported in tobacco [83]. Expression of *BjGDH2* was downregulated under 24h of osmotic and heat stress and returned to basal level at 24h of cold stress. An early upregulation of *BjGDH2* gene may have protective role in adjusting *B*. *juncea* plant under initial exposure to stress condition.

#### Asparagine synthetase

Asparagine synthetase (ASN) is an ATP-dependent enzyme that catalyses the transfer of the amide amino group of glutamine to a molecule of aspartate to generate glutamate and asparagine [84]. Increased asparagine level was reported under water deprivation and salinity stress conditions [85,86]. The accumulation of asparagine under stress is may be either due to increase in its biosynthesis or may be due to degradation of proteins. However, in *Coleus blumei*, ^14^C labeling experiment has suggested involvement of *de novo* synthesis in accumulation of free asparagines under salinity stress [86]. The sequence identity among ASN homologs ranged between 76–90.79% at amino acid level (S3 File). Expression of two genes encoding ASN was studied under different stresses, which revealed that expression of *BjASN1* was upregulated under salt (1h, 24h), osmotic (24h) and heat (24h) stresses, while it was and downregulated under cold (1h, 24h) and heat (1h) stresses (Fig 3E, S2 Fig). However, expression of *BjASN2* was found to be downregulated under all the stress conditions. Increased expression of *BjASN1* gene may lead to increased accumulation of asparagine under stress conditions. Under abiotic stress conditions, expression of *ASN1* was also reported to be induced in wheat seedlings, maize and sunflower [87–89].

## Conclusions

**Fig 4:**
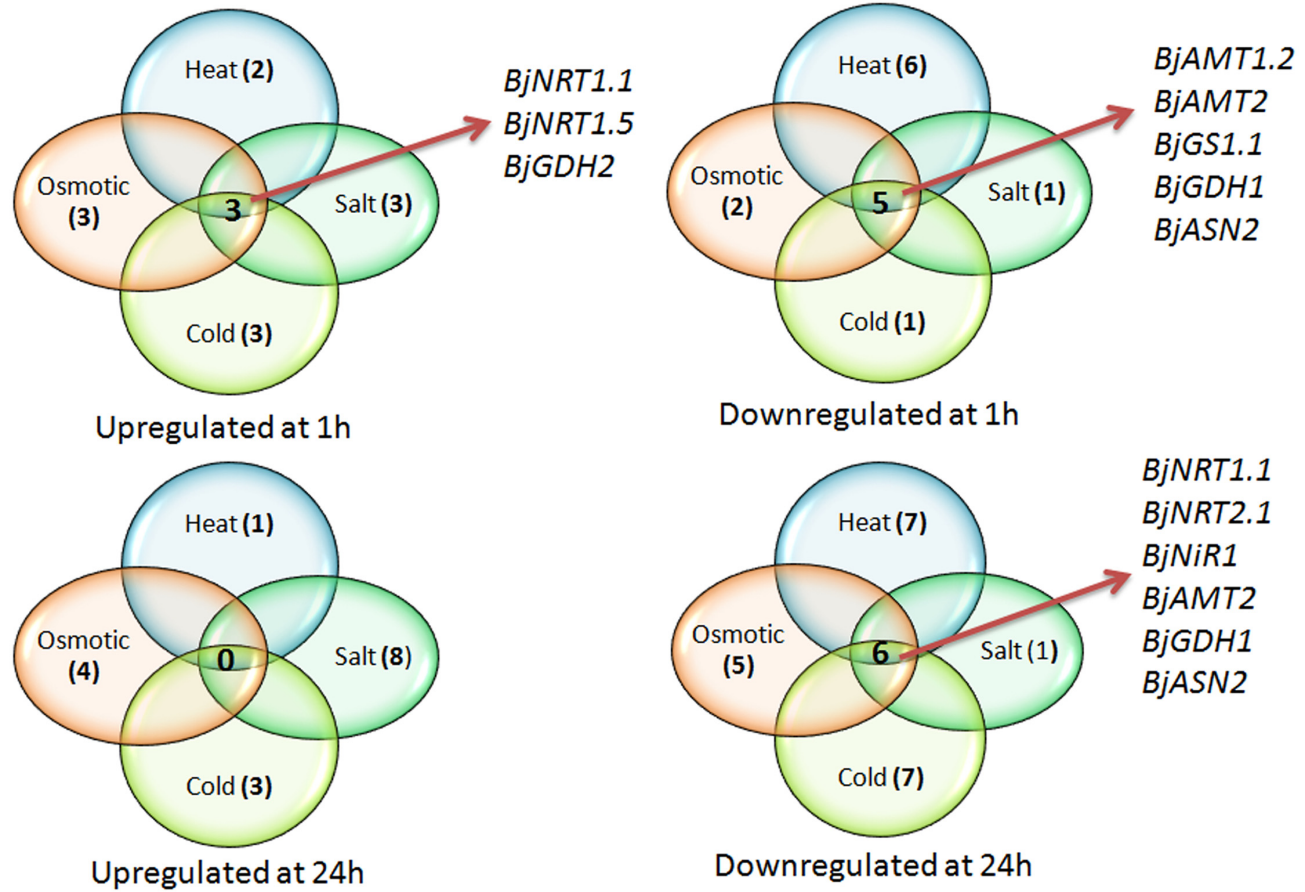
Venn diagram showing genes commonly upregulated and downregulated after 1h and 24h of stress treatments.

Abiotic stresses affect virtually all aspects of plant life, including nitrogen metabolism. However in case of *B*. *juncea*, effect of abiotic stress on nitrogen metabolism has been studied only at enzymatic level, so far. The present work represents the first report where effect of various abiotic stresses on expression of all the genes involved in Nitrogen transport, its reduction and its assimilation has been shown in any plant species. In order to understand the interaction of N pathway with abiotic stresses, expression profiling of N pathway genes was carried out under salt, osmotic, cold and heat stresses. We have cloned twenty six genes encoding various transporters involved in N uptake (NRT and AMT) and various enzymes involved in N assimilation (NR, NiR, GS, GOGAT, GDH and ASN) in *B*. *juncea*. Detailed expression profiling revealed that expression of various N pathway genes was modulated at different time points under various abiotic stress conditions. Differential expression pattern of genes, which encode for proteins involved in similar function may be due to the difference in regulatory motifs in their promoter regions. After 1h of stress treatments, three genes (*BjNRT1*.*1*, *BjNRT1*.*5* and *BjGDH2*) were found to be upregulated, whereas five genes (*BjAMT1*.*2*, *BjAMT2*, *BjGS1*.*1*, *BjGDH1*, and *BjASN2*) were found to be downregulated under all the stresses, commonly (Fig 4). However after 24h of stress treatments, no gene was found to be commonly upregulated, while expression of six genes (*BjNRT1*.*1*, *BjNRT2*.*1*, *BjAMT2*, *BjNiR1*, *BjGDH1* and *BjASN2*) was found to be commonly downregulated. Taken together, this analysis revealed that downregulation of *BjNRT1*.*1* and *BjNRT1*.*5*, which are considered important for nitrate uptake and translocation from root to shoot, respectively may lead to reduction in nitrate content in plant tissues. The reduced nitrate content in plant tissues may lead to downregulation of gene involved in N assimilation under stress conditions. Therefore, our analysis showed that longer exposure of abiotic stresses adversely affect all the processes of N pathway *viz*. N uptake, assimilation and mobilization in *B*. *juncea*. This may be one of the key components, which reduce plant growth and productivity under abiotic stresses. In conclusion, the present study provides an insight of regulation of various processes of genes of N pathway under abiotic stresses, which may help in selecting some of the genes of N uptake and assimilation pathway to develop transgenic plants with optimum yield potential under abiotic stress conditions.

## Supporting Information

S1 Fig Phylogenetic analyses of *B*. *juncea* protein sequences with *A*. *thaliana* orthologs. Phylogenetic trees of NRT1 (A), NRT2 (B), AMT (C), NR (D), NiR (E), GS (F), GOGAT (G), GDH (H) and ASN (I) protein sequences with respective *A*. *thaliana* orthologs were made using neighbour-joining method with 1000 bootstrap replicates.
(DOCX)

S2 Fig Heat Map showing relative expression of various genes encoding nitrate and ammonium transporters and enzymes involved in nitrogen assimilation in *B*. *juncea* under abiotic stress conditions after 1h (A) and 24h (B) as compared to untreated control plants. Relative expression ratios were determined using qRT-PCR. Bar at the bottom indicates relative expression ratios.
(DOCX)

S1 File List of primers used for amplification of genes. (XLSX)

S2 File List of primers used for qRT-PCR analysis and their amplicon size. (XLSX)

S3 File Percent sequence identity among homologs of *Brassica juncea* proteins. (XLSX)
