# Supplementary material for: Abiotic Stresses Downregulate Key Genes Involved in Nitrogen Uptake and Assimilation in Brassica juncea L
Source: PLoS One. 2015 Nov 25;10(11):e0143645. doi: 10.1371/journal.pone.0143645 (PMC4659633; doi:10.1371/journal.pone.0143645)
Supplement: S1 Fig — Phylogenetic trees of NRT1 (A), NRT2 (B), AMT (C), NR (D), NiR (E), GS (F), GOGAT (G), GDH (H) and ASN (I) protein sequences with respective A. thaliana orthologs were made using neighbour-joining method with 1000 bootstrap replicates. (DOCX) [file pone.0143645.s001.docx]

**S1 Fig.** **Phylogenetic analyses of *B. juncea* protein sequences with *A. thaliana* orthologs.** Phylogenetic trees of NRT1 (A), NRT2 (B), AMT (C), NR (D), NiR (E), GS (F), GOGAT (G), GDH (H) and ASN (I) protein sequences with respective *A. thaliana* orthologs were made using neighbour-joining method with 1000 bootstrap replicates.
