# Supplementary material for: Abiotic Stresses Downregulate Key Genes Involved in Nitrogen Uptake and Assimilation in Brassica juncea L
Source: PLoS One. 2015 Nov 25;10(11):e0143645. doi: 10.1371/journal.pone.0143645 (PMC4659633; doi:10.1371/journal.pone.0143645)
Supplement: S2 Fig — Relative expression ratios were determined using qRT-PCR. Bar at the bottom indicates relative expression ratios. (DOCX) [file pone.0143645.s002.docx]

**S2 Fig.** Heat Map showing relative expression of various genes encoding nitrate and ammonium transporters and enzymes involved in nitrogen assimilation in *B. juncea* under abiotic stress conditions after 1h (A) and 24h (B) as compared to untreated control plants. Relative expression ratios were determined using qRT-PCR. Bar at the bottom indicates relative expression ratios.
